# Effects of Qigong Exercise on Non-Motor Symptoms and Inflammatory Status in Parkinson’s Disease: A Protocol for a Randomized Controlled Trial

**DOI:** 10.3390/medicines6010013

**Published:** 2019-01-18

**Authors:** Sanghee Moon, Caio V. M. Sarmento, Irina V. Smirnova, Yvonne Colgrove, Kelly E. Lyons, Sue M. Lai, Wen Liu

**Affiliations:** 1 Department of Physical Therapy and Rehabilitation Science, School of Health Professions, University of Kansas Medical Center; 3901 Rainbow Blvd., MailStop 2002, Kansas City, KS 66160, USA; smoon@kumc.edu (S.M.); cmessiassarmento@kumc.edu (C.V.M.S.); ismirnova@kumc.edu (I.V.S.); ycolgrove@kumc.edu (Y.C.); 2 Department of Neurology, School of Medicine, University of Kansas Medical Center, 3901 Rainbow Blvd., MailStop 2012, Kansas City, KS 66160, USA; klyons@kumc.edu; 3 Department of Preventive Medicine and Public Health, School of Medicine, University of Kansas Medical Center, 3901 Rainbow Blvd., MailStop 1008, Kansas City, KS 66160, USA; slai@kumc.edu

**Keywords:** Parkinson’s disease, Qigong, mind-body therapies, cytokines, randomized controlled trial

## Abstract

**Background:** Non-motor symptoms such as sleep disturbance, cognitive decline, fatigue, anxiety, and depression in Parkinson’s disease (PD) impact quality of life. Increased levels of pro-inflammatory cytokines in individuals with PD have been reported, which may contribute to non-motor symptoms. A mind-body exercise, Qigong, has demonstrated benefits across different medical conditions. However, a lack of evidence causes clinicians and patients to be uncertain about the effects of Qigong in individuals with PD. This study will examine the effects of Qigong on non-motor symptoms and inflammatory status in individuals with PD. **Methods:** Sixty individuals with PD will be recruited. Qigong and sham Qigong group (n = 30 for each) will receive a 12-week intervention. Participants will practice their assigned exercise at home (2×/day) and attend routinely group exercise meetings. **Results:** Clinical questionnaires and neuropsychological tests will measure non-motor symptoms including sleep quality (primary outcome). Biomarker assays will measure inflammatory status. A two-way mixed-design analysis of variance (ANOVA) will be utilized. **Conclusions:** This study may generate evidence for the benefits of Qigong on non-motor symptoms of PD and the effect on inflammatory status. Findings may lead to the development of a novel, safe, and cost-effective rehabilitation approach for individuals with PD.

## 1. Introduction

Parkinson’s disease (PD) is a neurodegenerative disorder leading to both motor and non-motor symptoms. Motor symptoms, such as resting tremor, rigidity, bradykinesia, and postural instability, have traditionally drawn more attention from clinicians and researchers. Growing evidence suggests that non-motor symptoms can significantly impact quality of life, cause disability, and result in more health-related issues than motor symptoms [1,2,3]. Non-motor symptoms such as sleep disturbance, cognitive decline, fatigue, anxiety, and depression are commonly reported by individuals with PD [4]. A previous study indicated that 21% of individuals with PD reported non-motor symptoms in the early stage of PD, but this number increased to 88% seven years after diagnosis [5]. Sleep disturbances such as insomnia, restless leg syndrome, hypersomnia, and rapid eye movement sleep behavior disorder are particularly common in PD and reported to occur in 60% to 98% of individuals with PD [6]. As the disease progresses, individuals with PD show increased sleep latency and decreased total sleep time, deep sleep time, rapid eye movement sleep time, and sleep efficiency [7]. A common sleep disturbance in PD, rapid eye movement sleep behavior disorder, is associated with other non-motor symptoms including cognitive abnormalities [8,9]. Thus, improvement of sleep quality may help to increase quality of life and alleviate other symptoms in individuals with PD.

Neurodegeneration is caused by multiple contributors such as genetic, biological, and environmental factors [10]. Neuroinflammation, activated by microglial and immune cells in the brain, is one of the main contributors to neurodegeneration in PD [11]. Increased levels of neuroinflammatory biomarkers such as inflammatory cytokines in PD were reported in previous studies [12,13,14,15]. A postmortem PD brain study reported that the levels of interleukin (IL)-1β, IL-6, and tumor necrosis factor (TNF)-α were significantly increased in the cerebrospinal fluid [12]. In vivo, higher levels of IL-1β and TNF-α were found in individuals with PD compared to the healthy controls [13]. Other in vivo studies also showed increased levels of IL-6 and TNF-α in serum collected from individuals with PD [14,15].

Although it may be impossible to stop neurodegeneration in PD, many therapeutic approaches have been developed to alleviate symptoms in PD. Qigong is a mind-body exercise that has shown benefits on symptoms in PD. A study involving individuals with mild to moderate PD demonstrated a significant improvement in sleep quality [16]. In our feasibility study in a single-arm design (n = 7), a 6-week Qigong exercise program demonstrated an improvement in sleep quality measured by Parkinson’s Disease Sleep Scale-2 (PDSS-2), in which the results showed sub-scores of PDSS-2 were significantly improved or showed a trend of improvement including motor symptoms at night (*p* < 0.05) and disturbed sleep (*p* = 0.06) in individuals with PD [17]. Our recent pilot study, a randomized controlled trial (RCT), in individuals with PD showed a significant improvement in sleep quality, including PD symptoms at night (*p* < 0.05) and total PDSS-2 score (*p* < 0.001), after practicing the Qigong exercise for eight weeks (2-week training + 6-week Qigong exercise) [18].

The exact mechanism of Qigong exercise behind clinical improvements observed in patients with PD has not yet been completely understood. A possible mechanism of Qigong exercise may involve biological pathways related to neuroinflammation. The deep abdominal breathing may stimulate serotonin pathways by stimulating enterochromaffin cells that release serotonin [19] and interact with pro-inflammatory cytokines that contribute to neuroinflammation in the brain [20]. Our recent pilot study suggested potential benefits of Qigong exercise through changes in inflammation status, in which the results showed a significant decrease in serum level of TNF-α from 13.8 ± 0.6 to 12.3 ± 1.6 (*p* < 0.05) that was significantly correlated with changes in sleep quality and serum TNF-α level (R^2^ = 0.62) [18]. In various medical conditions, mind-body exercises such as Yoga, Tai Chi, and Qigong improved the immune system [21]. Particularly, past studies reported the benefits of Qigong exercise on immunological functions in healthy people [22] and individuals with cancers [23,24,25]. 

For the PD population, many studies were performed to investigate the effects of Qigong on motor symptoms in individuals with PD. However, to the best of our knowledge, only four RCTs have studied the effects of Qigong on non-motor symptoms in individuals with PD [16,18,26,27]. In those previous studies, although the results showed a potential of Qigong exercise to improve non-motor symptoms in PD, there was missing information such as rationale for sample size estimation, selection bias, attrition bias, and participant blindness. Moreover, past studies did not systematically explore the underlying mechanism of Qigong exercise for improving non-motor symptoms in PD, which may make clinicians hesitate to recommend Qigong exercise for their patients [28].

In this study protocol, we mainly discuss the rationale and research design of the study. The study aims to examine the efficacy of Qigong exercise on non-motor symptoms and neuroinflammatory status in PD. We hypothesized that Qigong exercise will improve non-motor symptoms such as sleep disturbance (primary outcome), cognitive decline, fatigue, pain, mood, and quality of life, and decrease serum levels of pro-inflammatory cytokines including IL-1β, IL-6, and TNF-α in individuals with PD. In addition, this study explores the relationship between changes in non-motor symptoms and changes in inflammatory status.

## 2. Study Design and Aims

### 2.1. Study Design

**Figure 1:**
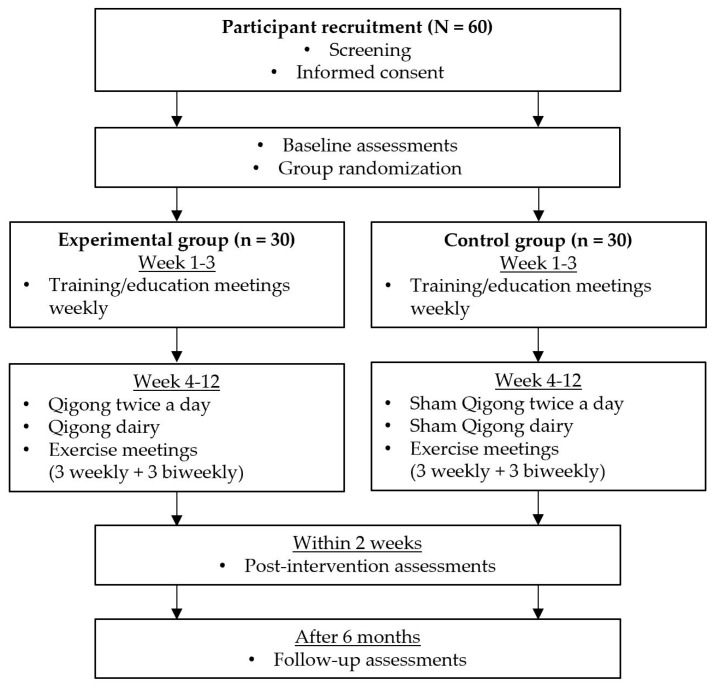
Study flow chart. A screening will be conducted using the screening tool developed by our research laboratory (Table A1). A study participation consent will be obtained and a baseline assessment will be conducted at the initial visit. After group randomization, participants will be assigned into either experimental or control groups. Throughout a 12-week intervention, participants will perform the intervention exercise twice a day at home and attend a total of 9 group meetings (weekly meeting for first 6 weeks and then biweekly meeting for next 6 weeks). After completing the 12-week intervention, a post-intervention assessment will be conducted within 2 weeks after the 12-week intervention. A follow-up assessment will be conducted 6 months after the completion of 12-week intervention.

We designed a double-blind RCT involving 60 individuals with PD (Figure 1). The participants will be randomly assigned to either an experimental (n = 30) or control (n = 30) group. Each group will have group exercise meetings (nine times in total; weekly meeting for first six weeks and then biweekly meetings for next six weeks) throughout the 12 weeks of the intervention period. Participants will be blinded to their group assignments until the end of the study. At baseline, all outcome measures and demographic variables including age, sex, ethnicity, years of education, disease duration, and medications will be collected. After the baseline assessments, participants in both groups will have a 12-week intervention period, including three weeks of training and nine weeks of exercise. The duration of the intervention will be longer than our two previous studies to provide more structured training sessions and longer intervention time [17,18]. During the training period, participants in the experimental group will learn the Qigong exercise, whereas those in the control group will learn the sham Qigong exercise. During the exercise period, participants will continue to practice what they will have learned during the training period. Throughout the 12-week intervention period, all participants will be asked to perform their assigned exercise (Qigong or sham Qigong exercise) twice a day at home, in the morning and evening, attend group meetings (weekly meeting for first six weeks and then biweekly meetings for next six weeks) and keep an exercise diary. The exercise diary includes daily checklists (morning exercise (yes/no), evening exercise (yes/no), and other exercise more than 0.5 h (yes/no)), subjective scales (sleep quality (1–5), fatigue level (0–10), and pain level (0–10)), and participant’s note for any unusual events. Within two weeks after the completion of the 12-week intervention, post-intervention assessments will be conducted, which are the same as the baseline assessments. The assessments will be completed again six months after the end of the intervention period.

### 2.2. Specific Aims

Specific aim I. To examine the effects of Qigong exercise on non-motor symptoms in PD.

Primary hypothesis: Participants will have greater improvement in sleep quality after Qigong exercise than the participants after sham Qigong exercise.

Secondary hypothesis I: Participants will have greater improvement in cognitive function, fatigue, anxiety, depression, overall non-motor symptoms, and quality of life after Qigong exercise than the participants after sham Qigong exercise.

Specific aim II. To examine changes in inflammatory biomarkers in serum after practicing Qigong exercise.

Secondary hypothesis II: Participants will have greater reduction in levels of inflammatory biomarkers (IL-1β, IL-6, and TNF-α) after Qigong exercise than the participants after sham Qigong exercise.

Specific aim III. To examine relationships between changes in non-motor symptom scores and changes in the inflammatory status in PD after the intervention (both Qigong and sham Qigong).

Secondary hypothesis III: Changes in non-motor symptom scores will significantly correlate with changes in levels of the inflammatory biomarkers.

### 2.3. Ethics Approval and Informed Consent

The study protocol and the consent form were approved by the Institutional Review Board of the University of Kansas Medical Center. The study ID is #STUDY00140835. This study is registered at www.clinicaltrails.gov (NCT03463330) [29].

### 2.4. Study Criteria

#### 2.4.1. Inclusion Criteria

The study inclusion criteria include (Table 1): (1) diagnosis of idiopathic PD according to the United Kingdom Parkinson’s Disease Brain Bank Society diagnostic criteria [30], (2) women and men aged 40 to 75 years, (3) currently taking levodopa with some improvement in motor symptoms, (4) levodopa dose has been stable for a minimum of four weeks prior to the start of the study, and (5) Hoehn and Yahr stage I to III (mild to moderate PD).

#### 2.4.2. Exclusion Criteria

The study exclusion criteria include (Table 1): (1) Mini Mental State Examination score <24 [31], (2) neurological diseases other than idiopathic PD including other forms of parkinsonism, uncontrolled or significant cardiovascular diseases, orthopedic, balance, or medical problems, (3) prior major head trauma with loss of consciousness, (4) deep brain stimulation, and (5) expected change in PD medications or non-motor symptom medications (e.g., sleep, anxiety, and depression medications) that may affect the results of the proposed outcome measures over the course of the study.

#### 2.4.3. Withdrawal/Termination Criteria

The withdrawal or termination criteria include: (1) death, (2) withdrawal of consent for any reason, (3) physician discretion, (4) major changes in PD medications or other non-motor symptom medications; and (5) failure to comply (due to hospitalizations, vacations, etc.) with prescribed exercise.

### 2.5. Recruitment and Screening

Participants will be recruited primarily from the Parkinson’s Disease and Movement Disorder Center at the University of Kansas Medical Center, in which approximately 200 new and 1400 returning individuals with PD are seen annually. Among individuals with PD being seen, we expect about two thirds will meet our recruitment criteria. Individuals who indicate an interest to participate in research will be contacted by a member of our team. Physician’s permission will be obtained prior to an individual’s participation in the study.

An initial screening will be conducted via phone by members of the research team using a screening script (Table A1).

### 2.6. Informed Consent

Members of the research team will give participants detailed and comprehensive information about the study and help participants to make an independent decision with regard to their participation in the study. Any potential participant will undergo a process of informed consent. If a participant agrees, he/she will sign a consent form approved by the Internal Review Board at the University of Kansas Medical Center. Throughout the process, members of the research team will avoid any coercion and ensure the participant’s confidentiality.

### 2.7. Randomization and Double-Blinding

All participants will be assigned a randomly generated 6-digit alphanumeric code after consenting to the study. Assignment to either experimental or control group will be determined by a computer-generated random code (e.g., 0 or 1) in a 1:1 ratio. All participants and outcome assessors will be blinded to the group assignment and intervention. Collected data will be stored on the secured server provided by the University of Kansas Medical Center. All individual information will be stored in the de-identifiable form.

## 3. Intervention

### 3.1. Experimental Group

The experimental group will learn a specific type of Qigong exercise named ‘six healing sounds’ Qigong. The six healing sounds Qigong exercise consists of mild-body movements, meditation, and abdominal breathing along with uttering six healing sounds. The detailed instruction for the body movements and sounds can be found in Table 2. This form of exercise is chosen because it is appropriate for individuals with PD who may have reduced physical capacity since this exercise is easy to learn and safe to perform. The exercise mainly involves upper mild-body movements and can be performed in any position including standing (preferred), sitting, or lying down. Trained Qigong instructors will lead the 12-week intervention period throughout the study. The format of the Qigong exercise program will be the same over the 12-week intervention.

Participants will be instructed to practice the Qigong exercise at home twice a day in the morning after getting up and in the evening before going to bed. A Qigong exercise session usually takes between 15 and 20 minutes to perform. Participants will be asked to record their exercise performance and self-rated levels of pain, fatigue, sleep quality in a daily exercise diary throughout the study. Participants will have a group meeting every week for the first six weeks and every other week for the remaining six weeks. During group meetings, the Qigong instructor will assess the performance of each participant and discuss relevant issues for about an hour. Also, if a participant shows a sign of low adherence to the exercise, the instructor will provide a behavioral coaching to increase the adherence level based on the model of behavioral change [32]. After the completion of the 12-week intervention period, participants will be asked to continue practicing the Qigong exercise twice a day at home and keep an exercise diary. At the end of the 6-month follow-up, participants will return for a follow-up assessment, which is the same as the baseline and post-intervention assessments.

### 3.2. Control Group

To match with the experimental group in terms of the intensity of activities and duration of the study, a sham Qigong exercise will be utilized. The sham Qigong instructor will be blinded to the study hypothesis to avoid a potential bias and minimize any influence on study outcomes. The sham Qigong exercise consists of the same mild body movements as the Qigong exercise. However, participants in the control group will not be taught abdominal breathing techniques with six healing sounds and meditation during the exercise. The format of Sham Qigong exercise program will be the same over the 12-week intervention. The control group will have group meetings separately from the experimental group, once per week in the first six weeks, and every other week the last six weeks. During group meetings, the sham Qigong instructor will assess the performance of each participant and discuss relevant issues that are the same topics for the experimental group meeting for about an hour. The same baseline, post-intervention, and 6-month follow-up assessments will be conducted. This group will be asked to practice the sham Qigong exercise twice a day at home and keep an exercise diary.

### 3.3. Adverse Events

Any adverse events will be immediately reported to the principal investigator and, if appropriate, to the Human Participants Committee per the University of Kansas Medical Center Human Participants Committee policies. The principal investigator will be notified immediately of chest pain, irregular pulse, fainting, shortness of breath, light headedness, dizziness, excessive sweating, slurred speech, blurred vision, leg swelling, falls, or any other adverse events.

## 4. Outcome Measures

### 4.1. Demographic and Clinical Information

Demographic information including age, date of birth, sex, race, occupation, highest level of education completed, and years at school from elementary. In addition, clinical information will be obtained including diagnosis, date of diagnosis, and current medications. Participants will be asked to report if there are any changes in medications during the study period.

### 4.2. Clinical Assessments

The following clinical questionnaires and neuropsychological tests, commonly used in research and clinical settings in individuals with PD, will be utilized to assess participants. Based on our previous trials, the total estimated time to complete the following clinical questionnaires is an hour.

- Sleep quality will be measured using the Parkinson’s Disease Sleep Scale-2 (PDSS-2). This 15-item questionnaire addresses issues related to sleep quality, which has been shown to be reliable in identifying issues specific to PD [33]. A lower score indicates better sleep quality. The minimal clinically important difference (MCID) is the threshold of −3.44 points for detecting improvement or the threshold of 2.07 points for detecting worsening [34].
- Cognitive function will be assessed using standardized clinical testing methods. The Frontal Assessment Battery evaluates executive function including conceptualization, mental flexibility, programming, sensitivity to interference, inhibitory control, and environmental autonomy [35]. Its maximum score is 18. A higher score indicates better performance. The ten-point clock test will be used to evaluate cognitive deficits. In this test, participants will draw a round-faced clock with hands at ten past eleven. Scoring will be performed using the criteria by Manos and Wu (1994) [36]. The trail making test parts A and B will assess visual attention and task switching. In these tests, participants will connect a set of dots as quickly as possible. The completion time will be compared with normative data [37].
- Comprehensive non-motor symptoms in PD will be measured by the Parkinson’s Disease Non-Motor Symptom Questionnaire [38]. The assessment contains a total of 30 items including 10 domains (gastrointestinal tract symptoms, urinary tract symptoms, sexual function, cardiovascular symptoms, apathy/attention/memory, hallucinations/delusions, depression/anxiety/anhedonia, sleep/fatigue, pain, and miscellaneous (diplopia, weight loss, etc.)). A lower total score indicates milder PD symptoms.
- Fatigue level will be measured by the 16-item Parkinson Fatigue Scale, which has shown reasonably good reliability and validity in testing fatigue specific to PD [39]. Its maximum score is 90. A lower score indicates better symptoms.
- Anxiety and depression will be measured using the 20-item Geriatric Anxiety Inventory and 15-item Geriatric Depression Scale [40,41]. For both scales, a lower score indicates less anxiety or depression.
- The severity of PD motor and non-motor symptoms will be assessed by the Movement Disorder Society—Unified Parkinson’s Disease Rating Scale (UPDRS) [42]. This assessment consists of four parts including non-motor experiences of daily living (13 items), motor experiences of daily living (13 items), motor examination (18 items), and motor complications (6 items). A lower score indicates milder PD symptoms. The MCID is 2.5 points for detecting minimal improvement in the motor score and 4.3 points for observing minimal improvement in the total score [43].
- Quality of life will be evaluated using the 39-item Parkinson’s Disease Questionnaire [44]. This assessment covers eight dimensions, including mobility, activities of daily living, emotional well-being, stigma, social support, cognition, communication, and bodily discomfort. A lower score indicates better quality of life. The MCID of sub- and overall-scores suggested by Peto et al. (2001) will be utilized [45].

Of note, the UPDRS is a standard measure of PD severity, which includes measurements of motor impairment and non-motor symptoms in individuals with PD. However, the primary focus of the study is non-motor symptoms, thus the motor symptoms of UPDRS will only be used to describe clinical status of the participants. The results of the questionnaires will be manually entered into an encrypted database by a member of research team who is blinded to study participants and group assignments.

### 4.3. Serum Biomarkers

Venous blood samples will be collected from participants three times, baseline, post-intervention, and 6-month follow-up. At each timepoint about 30 mL of blood will be collected using the BD vacutainer^®^ blood collection tubes (Becton, Dickinson and Company, Franklin Lakes, NJ, USA; catalog number 367820). Blood draws will be performed by a registered nurse between 9 am and 11 am. After the blood draw, collected samples will be placed at room temperature for approximately 30 minutes for coagulation. Coagulated blood samples will be centrifuged at 1,000 × g at 4 °C for 10 min to separate serum from blood clot and cells. Collected serum will be immediately aliquoted and stored at −80 °C in a freezer until assaying.

When performing the assay, aliquoted samples will be thawed, gently vortexed, and processed to separate any precipitation by centrifugation at 14,000 × g at 4 °C. We will measure the levels of the following biomarkers by enzyme-linked immunosorbent assay (ELISA) using commercially available kits: IL-1β (RayBiotech, Norcross, GA, USA; detection range 3 pg/mL to 1000 pg/mL), IL-6 (RayBiotech, Norcross, GA, USA; detection range 3 pg/mL to 1,000 pg/mL), and TNF-α (ThermoFisher Scientific, Rockford, IL, USA; detection range 15.6 pg/mL to 1,000 pg/mL).

## 5. Sample Size Estimation, Statistical Methods, and Reporting Results

### 5.1. Sample Size Estimation

A total of 60 participants will be recruited. The estimation of sample size was based on 80% statistical power regarding one of the primary variables, overall sleep quality (PDSS-2 total score). In our preliminary study, we observed a significant change after Qigong exercise in overall sleep quality by 11.5 points between two groups (experimental vs. control). A pooled standard deviation between two groups was 10.7. With a statistical power of 80% and a Type I error rate at 0.05, the estimated sample size was 14 for each group. Considering a potential dropout rate of 30%, the estimated sample size is 20 for each group. However, this sample size may not have enough statistical power for testing secondary hypotheses related to variables of other non-motor symptoms and/or serum biomarkers. Therefore, we propose to enroll 30 participants in each of the intervention arms.

### 5.2. Statistical Analysis

Descriptive statistics including mean, standard deviation, frequency, and percentage will be calculated. Mean changes between the three-time points (baseline, post-intervention, and 6-month follow-up) in the experimental and control groups will be examined by two-way mixed-design analysis of variance (ANOVA). If no significant interaction is observed, the main effect will be analyzed using two-tailed t-tests with Bonferroni correction for groups and repeated measures ANOVA for time. Correlation coefficients (r) will be utilized to find relationships between variables including non-motor symptoms and inflammatory biomarker levels. Duration of PD will be used as a covariate in the analysis. The level of significance is set at 0.05. In addition, sub-group analysis based on demographic (e.g., sex and age) or clinical (e.g., Hoehn and Yahr stage and disease duration) information will be conducted.

### 5.3. Reporting Results

Study results will be reported under the Consolidated Standards of Reporting Trials (CONSORT) guidelines.

## 6. Discussion

This study protocol is designed to investigate the efficacy of Qigong exercise on non-motor symptoms and inflammatory status in individuals with PD. Qigong exercise has shown benefits in a variety of medical conditions [17,24,46,47,48,49]. However, to our knowledge, only a few studies, including our own, have examined the efficacy or effectiveness of Qigong exercise in improving non-motor symptoms such as sleep disturbance, cognitive decline, fatigue, pain, mood, and quality of life, as well as inflammatory status in the PD population [16,17,18,26,27].

Complementary and alternative medicine originating outside of conventional Western medicine is generally well-received among individuals with various medical conditions as well as healthy people. Particularly, the use of a mind-body intervention including yoga, Tai Chi, and Qigong gradually increased from 2002 to 2012, according to the United States national health statistics reports [50]. Despite that many people have benefited by complementary and alternative medicine, a lack of clinical research is one of the largest barriers for the clinical use of complementary approaches.

This study will offer a foundation for a future multi-center RCT to identify the effectiveness of Qigong exercise and will contribute to the development of a novel, safe, and cost-effective approach for individuals with PD. In addition, the study will examine the changes in inflammatory status induced by Qigong exercise in PD, providing a better understanding of the role of inflammation in PD, and will uncover the relationship between non-motor symptoms and inflammatory status in PD.

## Figures and Tables

**Table 1:** Inclusion and exclusion criteria and rationale.

Criteria	Rationale
Inclusion Criteria	
Idiopathic PD	Individuals who had been diagnosed based on the United Kingdom Parkinson’s Disease Brain Bank Society diagnostic criteria are included to establish a diagnostic baseline.
Women and men aged 40 to 75 years	Clinically, PD is not common in young individuals. In addition, individuals older than 75 years are more likely to have comorbidities or be on an advanced stage of PD, which may confound the study results.
Current use of levodopa	Levodopa is a standard medication for PD.
Stable levodopa dose	Changes in levodopa dosage may cause difficulties to determine whether the expected benefits are caused by the changed medication dose or the intervention.
Hoehn and Yahr stage I to III	Hoehn and Yahr stage above III involves significant motor impairment, which may restrict individual’s movement during exercise.
Exclusion Criteria	
Mini Mental State Examination <24	Less than score 24 on Mini Mental State Examination indicates cognitive impairment. Those individuals scored under 24 may have difficulties to follow study instructions.
Other neurological disease including other forms of parkinsonism, uncontrolled or significant cardiovascular diseases, orthopedic or medical problems	Other neurological conditions may confound the study results. Uncontrolled or significant cardiovascular conditions and orthopedic conditions may cause unwanted events during the exercise.
History of major head trauma with loss of consciousness	Qigong involves meditation. Individuals with head related injuries may have difficulties in meditation.
Deep brain stimulation	It is possible that deep brain stimulation alters mechanisms of PD symptoms compared with those without deep brain stimulation, which may affect the study results.
Expected change in PD medications or non-motor symptom medications (e.g., sleep, anxiety, and depression medications)	Changes in medication can induce changes in PD symptoms, which may confound the study findings.
Abbreviation: PD, Parkinson’s disease.	

**Table 2:** Six healing sounds Qigong exercise instruction

Movement	Sound	Body Movement and Breathing
Relaxation movement	No sound	When inhaling, lift up both arms/hands with elbows fully extended out from both sides with palms down. Lift arms to the shoulder level. Move both arms/hands horizontally to the front and then towards the chest. Exhale as arms/hands move down slowly until the end of exhalation. Repeat the breath and body movement three times. Perform this movement before Movement 1, between each movement, and after Movement 6 (a total of seven times throughout the exercise).
Movement 1	Hsu [shh]	When inhaling, lift up both arms/hands near the body to chest level, palms facing up. Then straighten the arms out to the sides. Then move the hands to the chest. Exhale as the arms/hands move down. During the slow exhalation, chant “shh”. Repeat the sound and movement six times.
Movement 2	Her [her]	When inhaling, lift up both arms/hands near the body to the chest level with the palms facing up. Begin to exhale. During exhalation, chant “her” and continue to slowly move arms/hands up to the eyebrow level. Inhale while moving arms/hands down. Convert to exhalation when hands pass the chest level and continue to move arms/hands down. Repeat the sound and movement six times.
Movement 3	Hoo [who]	When inhaling, lift up both arms/hands near the body to chest level, palms facing up. Then, begin to exhale and chant “who” while slowly moving your left hand up and right hand down in a diagonal direction until the end of exhalation. Inhale and move left hand down and right hand up to the chest level again. Convert to exhalation and chant “who” while slowly moving the left hand down and the right hand up in a diagonal direction until the end of exhalation. Repeat the sound and movement three times.
Movement 4	Sss [sss]	When inhaling, lift up both arms/hands to the chest level, palms facing up. Begin to exhale. During exhalation, chant “sss” while slowly pushing the hands forward and then down to both sides until the end of exhalation. Repeat the sound and movement six times.
Movement 5	Chway [ch-way]	When inhaling, lift up both arms/hands through the back of trunk to the front of the chest as if holding a large ball. Begin to exhale. During exhalation, chant “chway” while slowly moving both hands out over and down an imaginary ball until touching the thighs. (Bend both knees down slightly while you circle your hands down over the ball). Repeat the sound and movement six times.
Movement 6	See [see]	When inhaling, lift up both arms/hands near the body to the chest level, palms facing up. Begin to exhale. During exhalation, chant “see” (with a smile on your face) and continue to slowly lift hands straight over the head until the end of exhalation. Begin to inhale while slowly moving returning arms/hands along the same path. Begin to exhale again when the hands pass over the chest and continue to move arms/hands down until the end of exhalation. Repeat the sound and movement six times.

Throughout the entire exercise sequence, focus your mind on an important focal point, the so-called “Dan Tian” acupuncture point, which is located in the abdomen three finger widths below your belly button to establish and maintain the mind emptiness status. Note: The format of the exercise program will be the same over the 12-week intervention. The instruction is adapted from Moon et al. (2017).

## Appendix A

**Table A1:** Phone screening script.

Date:	
Subject Name:	
1. Assuming patient is available for answering questions, ask the following questions:	
2. Are you able to participate in a study which requires the practice of mild daily exercise and **about 11** visits to KU Medical Center over the course of 14 weeks? Specifically there will be 2 visits for assessment prior to and immediately following the study, 9 visits of group exercise sessions during the study. Will this work for you? **YES**—move onto question #3 **NO**—thank them for their time and let them know they are not eligible for this study at this time	
3. Have you been diagnosed with primary (or idiopathic) Parkinson’s disease? **YES**—move onto next question **NO**—thank them for their time and let them know they are not eligible for this study at this time	
4. Are you between the ages of 40 and 75? **YES**—move onto next question **NO**—thank them for their time and let them know they are not eligible for this study at this time	
5. Treatments a) Are you aware of any intentions of changing your medication or dosages over the next three months? **YES** **NO** b) Does your treatment include Deep Brain Stimulation (DBS)? **YES** **NO** **Part a) or Part b): YES**—thank them for their time and let them know they are not eligible for this study at this time **NO**—move onto next question	
6. Have you been diagnosed with any other medical problems (neurological, orthopedic, etc.) that interfere with your ability to do mild body movements and/or follow instructions? **YES**—thank them for their time and let them know they are not eligible for this study at this time **NO**—move onto next question	
7. Are you able to give a simple blood test which includes 30ml (about 1 oz.) of venous blood collection at pre- and post-testing? **YES**—move onto next question **NO**—thank them for their time and let them know they are not eligible for this study at this time	
8. Can you be contacted for participation in the study within two weeks? **YES**—move onto next question **NO**—When will you be available for the study ___________________	
9. What is the phone # and email address to contact you in the next three months? Cell phone # (if available): Email address:	
